# Evolutionary Tracking of SARS-CoV-2 Genetic Variants Highlights an Intricate Balance of Stabilizing and Destabilizing Mutations

**DOI:** 10.1128/mBio.01188-21

**Published:** 2021-07-20

**Authors:** Jobin John Jacob, Karthick Vasudevan, Agila Kumari Pragasam, Karthik Gunasekaran, Balaji Veeraraghavan, Ankur Mutreja

**Affiliations:** a Department of Clinical Microbiology, Christian Medical College, Vellore, India; b Department of General Medicine (Unit-V), Christian Medical College, Vellore, India; c Cambridge Institute of Therapeutic Immunology & Infectious Disease (CITIID) Department of Medicine, University of Cambridge, Cambridge, United Kingdom; d Department of Biotechnology, School of Applied Sciences, REVA University, Bangalore, India; University of Kansas Medical Center; KUMC

**Keywords:** SARS-CoV-2, mutation, evolution, stability, vaccine

## Abstract

The currently ongoing COVID-19 pandemic caused by SARS-CoV-2 has accounted for millions of infections and deaths across the globe. Genome sequences of SARS-CoV-2 are being published daily in public databases and the availability of these genome data sets has allowed unprecedented access to the mutational patterns of SARS-CoV-2 evolution. We made use of the same genomic information for conducting phylogenetic analysis and identifying lineage-specific mutations. The catalogued lineage-defining mutations were analyzed for their stabilizing or destabilizing impact on viral proteins. We recorded persistence of D614G, S477N, A222V, and V1176F variants and a global expansion of the PANGOLIN variant B.1. In addition, a retention of Q57H (B.1.X), R203K/G204R (B.1.1.X), T85I (B.1.2-B.1.3), G15S+T428I (C.X), and I120F (D.X) variations was observed. Overall, we recorded a striking balance between stabilizing and destabilizing mutations, therefore leading to well-maintained protein structures. With selection pressures in the form of newly developed vaccines and therapeutics to mount in the coming months, the task of mapping viral mutations and recording their impact on key viral proteins should be crucial to preemptively catch any escape mechanism for which SARS-CoV-2 may evolve.

## INTRODUCTION

The emergence of severe acute respiratory syndrome coronavirus 2 (SARS-CoV-2) in Wuhan, China and the subsequent global spread has brought the world to a standstill (1). During the course of 11 months, the coronavirus disease 19 (COVID-19) pandemic has caused more than 81 million confirmed cases in 220 countries, with close to 1,770,000 fatalities. (2). Initially, and rightly, the efforts were focused on minimizing the number of cases and deaths due to COVID-19 (3). This included fast tracking the search and development of novel treatment and prevention options (4). Today, however, as vaccine candidates have started showing promising results, there is a cautious shift towards assessing the efficacy of vaccine candidates with respect to the circulating diversity of SARS-CoV-2 and its continuously evolving genetic variants (5).

Functional mutations that help the virus to adapt to the recent host-shift events are hypothesized to drive the evolution of transmissibility and virulence in SARS-CoV-2 (6). Shortly after the first isolated SARS-CoV-2 genome from China was published, >30,500 distinct mutations were catalogued in the CoV-GLUE database (http://cov-glue.cvr.gla.ac.uk/) among globally circulating strains of this virus (7). Variations in the genetic makeup are key determinants in measuring the evolutionary distance and stability of SARS-CoV-2 from the first sequenced isolate (8). Moreover, tracking the evolution of SARS-CoV-2 since its introduction in humans is a high-priority undertaking to prevent future waves of this pandemic from escaping the global preparedness (9). Since many vaccine candidates currently under development are derived from the first available SARS-CoV-2 sequences, recurrent genetic changes may have an unforeseen impact on their sustained effectiveness in the longer term (10).

The availability of whole-genome sequences of SARS-CoV-2 in public repositories such as Global Initiative on Sharing All Influenza Data (GISAID) and real-time data visualization pipeline NextStrain (https://nextstrain.org) offers a great opportunity for scientists to track the evolutionary path of this virus (11, 12). Phylogenetic Assignment of Named Global Outbreak LINeages tool (PANGOLIN) has been the most widely used tool for lineage assignment to newly emerging variants. PANGOLIN (https://cov-lineages.org/pangolin.html) has also been deployed in establishing the transmission patterns of various clones of this virus (13). Since coronaviruses frequently recombine, tracking the evolution and assigning lineages has been challenging (13, 14). As a result, multiple studies that tracked the evolution of SARS-CoV-2 have been hugely controversial. For example, doubts have been cast on the claim of finding more aggressive L type strains emerging from S type strains (14). Similarly, the hypothesis that rapid spread of the D614G variant of SARS-CoV-2 indicates a possible fitness advantage has been questioned (15–17). Therefore, in the current and highly sensitive global circumstances due to this pandemic, having a detailed map of mutations highlighting their prospective role in therapeutics and vaccine development can prepare us better for the future waves of continuously evolving SARS-CoV-2. In this study, we present a catalogue of the most important genomic mutations recorded between December 2019 and November 2020 in SARS-Cov-2 and their possible impact on the stability of protein candidates that form the most crucial part of vaccines and also constitute the most common therapeutic targets.

## RESULTS

### Diversity of SARS-CoV-2 genomes.

**FIG 1:**
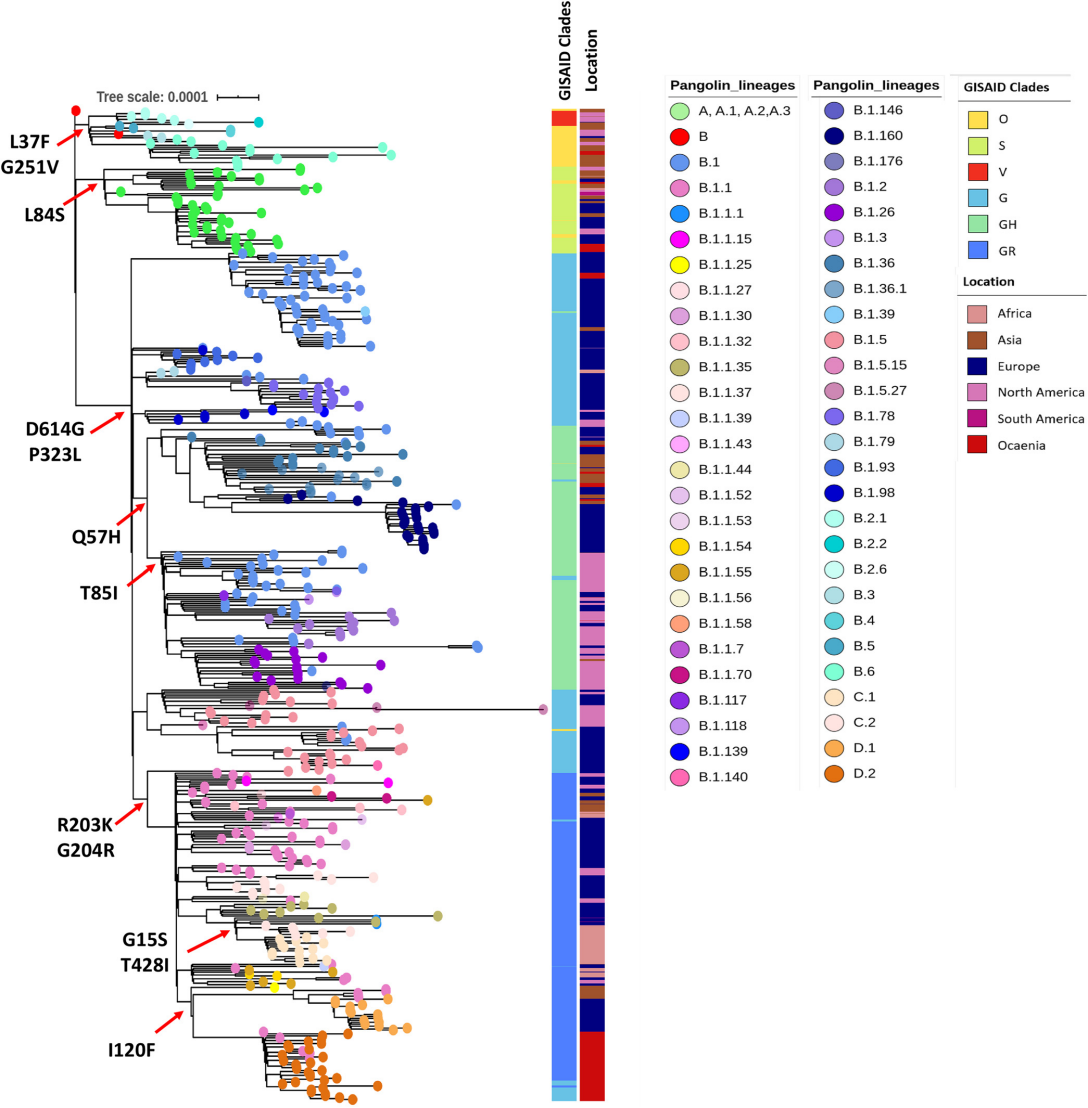
Maximum likelihood phylogenetic tree inferred from 513 SARS-CoV-2 genomes. The tree was constructed using multiple genome sequence alignment (MAFFT) by mapping against the Wuhan-Hu-1 strain (accession NC_045512). Tips are colored with the major lineages assigned by PANGOLIN. Respective lineages assigned by GISAID and origin of sequence are labeled as color strips. The scale bar indicates the distance corresponding to substitution per site.

Of the 7,000 SARS-CoV-2 genomes screened, we constructed a robust phylogenetic tree of 513 genomes strategically selected to reflect the most complete diversity among the isolates by covering all the PANGOLIN lineages. Lineage assignment based on the PANGOLIN tool indicated the circulation of seven distinct lineages and/or sublineages, such as A, B.1, B.1.1, B.1.1.1, B.2, B.3, and B.6. This is in line with the phylogenetic groupings by GISAID (S, L, V, O, G, GH, and GR) (Fig. 1). As the epidemic has progressed and mutations have accumulated, further subdivision of major lineages into sublineages has been observed. Overall, a total of 61 lineages and sublineages have been found to be circulating concurrently in multiple countries around the world. In general, numerous introductions of different variants were observed across the globe with a few sublineages (C.2, D.2) being restricted to certain regions. While the B.1.113 lineage, for example, has been exclusively reported from India, lineages C.2 and D.2 have been geographically confined to South Africa and Australia, respectively.

### Major amino acid substitutions.

**FIG 2:**
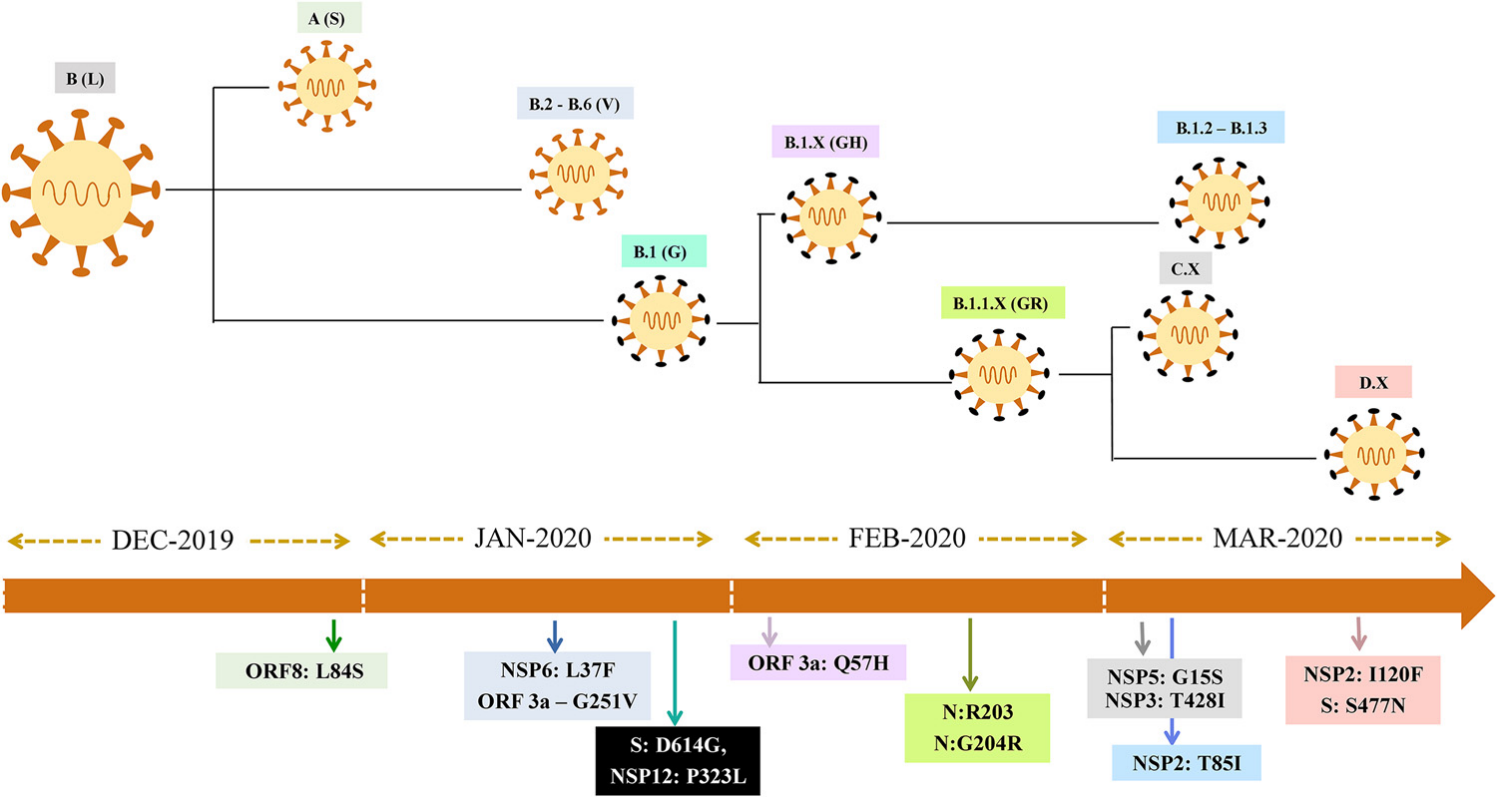
Schematic representation of the major evolutionary events/amino acid substitutions that gave rise to SARS-CoV-2 variants in sequential order.

Mutation mapping showed a total of 106 amino acid substitutions (missense mutations in >5 genomes) from a representative set of 513 genomes. The analysis also revealed 36 mutations that were found in >5% of genome sequences, while 12 major substitutions were lineage-defining mutations (Fig. 1). The first major mutation to appear was L84S in ORF8 (present in 8.6% of the genomes) that has defined the A lineage (i.e., clade S in the GISAID classification). The subsequent amino acid substitutions L37F in ORF3a and G251V in nsp6 were found to be present in 13.3% and 1.4% of genomes, respectively. The combination of G251V and L37F, which was initially considered a defining mutation pattern for the B.2 to B.6 lineage (clade V in GISAID classification), has shown under more detailed analysis that isolates carrying the G251V mutation are distributed in other lineages too. The predominant lineage-defining mutations in the whole data set were D614G (85.5%) and P323L (85.5%), after originally appearing in late January 2020 (Fig. 2). Other major mutations noted are Q57H (26.5%), R203K/G204R (33%), G15S (12%), I120F (11.5%), and T85I (14%).

### Dominance of the D614G variant.

Two mutations have become consensus: D614G in S (nucleotide 23,403, A to G) and P323L (also known as P4715L) in nsp12 (nucleotide 14,143, C to T). These mutations were present in 80.5% of the sequences and have defined the B.1 lineage (G in GISAID classification). The widely discussed D614G variant is speculated to have been introduced in Europe at the end of January (EPI_ISl_422424) before becoming globally dominant. Genomes with D614G mutations were assigned as B.1 by PANGOLIN or GH/GR by GISAID. Notably, founder lineage B.1 and its sublineages B.1.X, B.1.1.X, D.X, and C.X that carry both D614G and P323L mutations have become the dominant variants across the world (87% of global collection per CoV-GLUE as of 30 November 2020).

As the pandemic has progressed, several other major substitutions affecting the protein structure have appeared. These are Q57H (nucleotide 25,563, G to T) in ORF3a, the R203K + G204R combination (nucleotide 28,881, GGG to AAC) in nucleocapsid, and T85I (nucleotide 1,059, C to T) in ORF1a. The region-specific sub lineages C.1, C.2, D.1, and D.2 were found to cumulatively harbor multiple mutations. Amino acid substitutions such as T428I and G15S in ORF1a were reported in sublineages C.1 and C.2, and the S477N substitution in the spike (S) protein along with I120F in nsp2 specifically established the sublineage D.2 (Fig. 1).

### Structural analysis of SARS-CoV-2 mutants.

The possible structural consequences of 11 lineage-defining missense mutations identified in this study were investigated. Among the mutations, three were considered stabilizing to the respective protein structures, while six mutations were destabilizing (Table 1). The significance of these mutations in evolutionary selection cannot be solely predicted by ΔΔG, or change in free energy. Hence for a precise interpretation, correlation of ΔΔG, ΔΔS, and N-H S^2^ (Table S2 in the supplemental material) order parameter values of the proteins have been taken into account based on fine local alterations in structures. All lineage-defining mutations except two have reduced the vibrational entropies of the proteins, thereby decreasing the flexibility in the structures (Table 1).

**TABLE 1:** Lineage-defining SNPs and their impact on protein structures

Protein	Lineage-defining mutation	ΔΔS in kcal mol^−1^ K^−1^	Change in dynamics	ΔΔG in kcal mol^−1^ (DUET)	ΔΔG in kcal mol^−1^ (SDM)	Stability
Nsp12	P323L	−0.33	Decreasing flexibility	0.43 (stabilizing)	1.57 (stabilizing)	Stabilizing
Spike	D614G	−0.01	Decreasing flexibility	0.46 (stabilizing)	2.33 (stabilizing)	Stabilizing
Orf3a	G251V	−0.39	Decreasing flexibility	−0.6 (destabilizing)	−2.19 (destabilizing)	Destabilizing
	Q57H	0.44	Increasing flexibility	−1.25 (destabilizing)	0.87 (stabilizing)	Inconclusive
Orf8	L84S	0.30	Increasing flexibility	−1.41 (destabilizing)	−1.41 (destabilizing)	Destabilizing
Nsp2	T85I	0.07	Increasing flexibility	0.54 (stabilizing)	1.93 (stabilizing)	Stabilizing
	I120F	−1.30	Decreasing flexibility	−1.04 (destabilizing)	−0.21 (destabilizing)	Destabilizing
Nsp6	L37F	−0.29	Decreasing flexibility	−0.72 (Destabilizing)	−0.04 (neutral)	Inconclusive
Nucleocapsid	R203K	−0.98	Decreasing flexibility	−1.57 (destabilizing)	−0.48 (destabilizing)	Destabilizing
protein (N)	G204R	−0.16	Decreasing flexibility	−1.06 (destabilizing)	−1.95 (destabilizing)	Destabilizing
Nsp5	G15S	−0.31	Decreasing flexibility	−0.98 (destabilizing)	−0.79 (destabilizing)	Destabilizing

Additionally, the impact of mutations in key structural proteins that potentially allows any pathogen to escape available treatment and prevention regime was investigated. Among the 59 major missense mutations, our analysis using both the SDM and DUET servers predicted 16 missense mutations as stabilizing and 23 missense mutations as destabilizing the protein structure. Twenty major mutations were predicted to be neither stabilizing nor destabilizing, as the ΔΔG values provided by the SDM and DUET servers were contradictory (Table 2).

**TABLE 2:** Predicted effect of protein stability in the presence of amino acid mutations in the SARS-COV-2 genomes

Protein	Mutation	ΔΔG SDM (kcal/mol)	ΔΔG DUET (kcal/mol)	Stability
Spike	A222V	0.95	0.91	Stabilizing
	S477N	0.31	0.02	Stabilizing
	L18F	−0.801	−0.46	Destabilizing
	N439K	−0.29	0.3	Inconclusive
	L5F	−0.801	−0.1	Destabilizing
	W1214G	−1.913	−0.28	Destabilizing
	R21I	−0.856	0.46	Inconclusive
	A262S	−2.13	−1.66	Destabilizing
	S98F	1.23	−0.58	Inconclusive
	D1163Y	0.21	0.26	Stabilizing
	G1167V	−0.58	−2.25	Destabilizing
	D936Y	−0.13	−0.32	Destabilizing
	P272L	2.12	0.36	Stabilizing
	D80Y	0.77	−3.08	Inconclusive
	E583D	−0.69	−0.86	Destabilizing
	P1263L	−0.231	1.29	Inconclusive
	K1073N	−0.48	−0.45	Destabilizing
	D253G	−0.19	0.04	Inconclusive
	T723I	0.64	0.21	Stabilizing
	A688V	−0.18	0.03	Inconclusive
	A626S	−2.6	−1.66	Destabilizing
	L54F	−0.61	−1.33	Destabilizing
	H655Y	0.6	1.44	Stabilizing
	G769V	0.6	0.14	Stabilizing
	L176F	0.12	−0.95	Inconclusive
	G1124V	−1.52	−0.14	Destabilizing
	V622F	−0.2	−0.67	Destabilizing
	S255F	0.94	−0.8	Inconclusive
	H49Y	0.4	1.14	Stabilizing
	D839Y	−0.389	−1.08	Destabilizing
	V1176F	−0.92	−0.55	Destabilizing
	D215H	0.8	1.35	Stabilizing
	H146Y	1.139	−0.29	Inconclusive
	A879S	−2.57	−1.69	Destabilizing
	Q677H	0.98	−0.48	Inconclusive
	D1084Y	−0.43	−0.03	Destabilizing
	V1068F	−1.05	−1.15	Destabilizing
	P25S	−0.392	0.93	Inconclusive
	A520S	−1.23	−0.15	Destabilizing
	G261V	0.16	0.16	Stabilizing
	D574Y	−0.56	−0.45	Destabilizing
	T29I	0.48	0.51	Stabilizing
	Y453F	−0.17	−0.48	Destabilizing
	N501Y	0.41	−0.42	Inconclusive
	S939F	0.76	−0.71	Inconclusive
	T95I	1.91	0.37	Stabilizing
	Q675H	0.8	−0.4	Inconclusive
Nucleocapsid	A220V	−0.51	−1.13	Destabilizing
	S194L	1.15	−0.02	Inconclusive
	D103Y	1.45	0.55	Stabilizing
	P13L	0.84	0.23	Stabilizing
	S197L	1.27	0.26	Stabilizing
	A398V	−0.98	−1.03	Destabilizing
	P199L	1.27	−0.21	Inconclusive
	M234I	0.69	0.41	Stabilizing
	S188L	1.21	−0.06	Inconclusive
	S183Y	0.05	−0.71	Inconclusive
Membrane	T175M	0.69	−0.26	Inconclusive
Envelope	P71S	−0.03	−2.35	Destabilizing

### Balance of stabilizing and destabilizing mutations.

Overall, from both the data sets, 70 amino acid substitutions in SARS-CoV-2 were tested for stability, of which 19 were stabilizing, 29 were destabilizing, and 22 showed inconclusive results. Computational prediction to understand the effect of amino acid substitutions in SARS-CoV-2 revealed a balance of stabilization and destabilization of the proteins.

**FIG 3:**
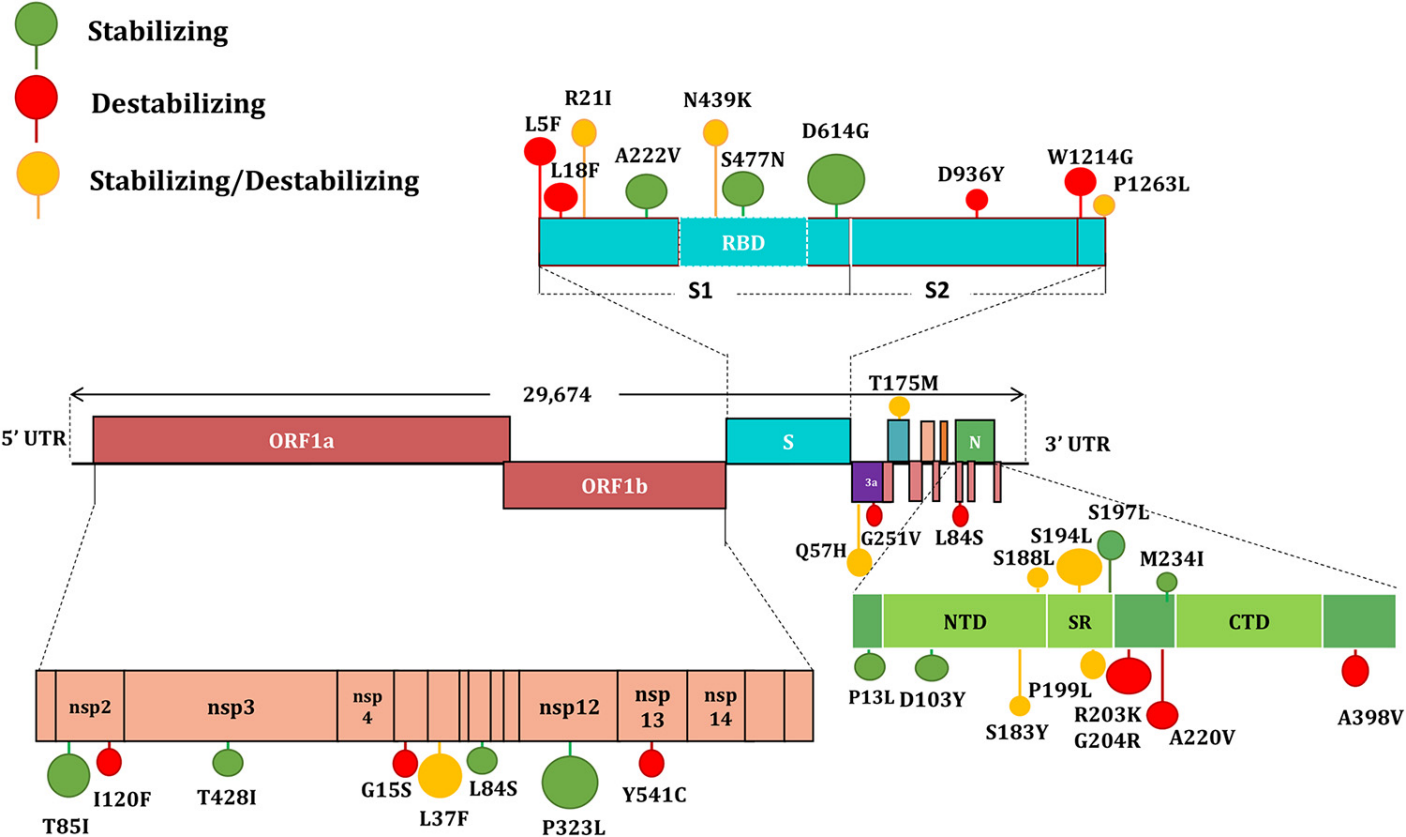
Schematic representation of SARS-CoV-2 genome organization, the major amino acid substitutions, and stability of amino acid changes. Stabilizing mutations are colored green, destabilizing mutations are colored red, and mutations that neither stabilize nor destabilize are colored yellow.

When checked for amino acid substitutions, the stabilizing mutation in spike (S) protein predicted an increase in the rigidity of its structure (Fig. 3; Fig. S1). The increased rigidities of the structure may provide a stable conformation to the protein that may positively influence the binding of spike protein to the ACE2 receptor (18). The major mutations D614G and S477N were located at potential epitope regions (codons 469 to 882), with S477N particularly positioned in the receptor-binding domain (RBD) of the S protein (319 to 541).

The most frequent amino acid substitutions were observed in the nucleocapsid (N) protein, in which the variants S194L, D103Y, P13L, S197L, M234I, and S188L were predicted to be stabilizing according to both the analytical servers (Table 2). In contrast, membrane (M) and envelope (E) proteins accounted for the least number of amino acid substitutions. The amino acid changes in M (T175M) indicated a stabilizing effect, while E does not account for any stabilizing variant. Structural analysis of double (D614G + S477N; D614G + A222V) and triple (D614G + S477N + A222V) mutation patterns in the S protein indicated ΔΔG values of 0.228, 0.195 and 0.129, respectively (Table 3). This signifies that accumulation of spike mutation in D614G-bearing lineages could potentially be affecting the stability of the spike and therefore may influence the binding affinity toward the ACE2 receptor.

**TABLE 3:** Impact of independent, double and triple mutations in the spike protein

Protein	Combinations	Mutations	ΔΔG (pred)	C (pred)
Spike	Independent	D614G	0.422	0.892
	Double	D614G+S477N	0.228	0.896
		D614G+A222V	0.195	0.889
	Triple	D614G+S477N+A222V	0.129	0.129

## DISCUSSION

Since the beginning of the COVID-19 pandemic, whole-genome, sequence-based phylogenetic inference has been heavily utilized in tracing viral origins and transmission chains (19). However, as the virus has evolved with time, genomic data are being increasingly used in guiding infection risk and control strategies. Several genomic mutations have been mapped that seem to be of advantage to the virus (20). In parallel, numerous vaccine candidates have been designed using genomic data from the original SARS-CoV-2 strain of Wuhan and many are now approved for use or at late-stage trials (21, 22). Based on immunological data obtained from infected and recovered patients, almost all COVID-19 vaccine candidates of today are based on the original SARS-CoV-2 spike protein or its RBD domain (23–25). However, as vaccines are introduced and successful treatment options become available, it is vital that we carefully monitor the mutations in the immunogenic region of SARS-CoV-2 genome (26). Mapping these changes to protein structure will allow preemptive forecasting of the direction of change in vaccine effectiveness and guide future preparedness efforts. We analyzed the impact of recurrent amino acid replacements in the genomic evolution and proteome stability of SARS-CoV-2 from its introduction in December 2019 through to November 2020. Our analysis found an intriguing balance of stabilizing and destabilizing mutations, which may have allowed SARS-CoV-2 to evolve and persist without losing pathogenicity.

SARS-CoV-2 is considered a slowly evolving virus, as it possesses an inherent proofreading mechanism to repair the mismatches during its replication. This is believed to have a crucial role in maintaining the stability and integrity of the viral genome (27, 28). Our analysis confirmed previously recorded positive natural selection of the D614G, S477N (29), A222V, and V1176F (30) variants and a global expansion of the PANGOLIN variant B.1 (11). In addition, we also observed a positive natural selection of Q57H (B.1.X), R203K/G204R (B.1.1.X), T85I (B.1.2-B.1.3), G15S+T428I (C.X), and I120F (D.X) variants (Fig. 2).

Apart from the 11 clade-defining mutations, some of the major missense mutations were in the four structural proteins (E, M, N, and S). When analyzed for their impact (*n* = 59) in the respective protein structure, the spike glycoprotein, more specifically its RBD domain, was found to be most vulnerable to frequent mutations. This may be due to the immunological observation that most neutralizing anti-SARS-CoV-2 antibodies have been found to target the RBD domain of the S protein (31, 32). Consistent with this finding, a total of 4,170 missense mutations have been reported in the spike protein, with 683 on the RBD domain alone (CoV-Glue, accessed 12 December 2020). Computational prediction to understand the effect of amino acid substitutions in E, M, N, and S proteins revealed a balance of stabilization and destabilization of the proteins. While viral populations carrying mutations with higher stabilizing effects (positive ΔΔG values) would be expected to become dominant variants, it is interesting to note that destabilization mutations in the major protein targets of SARS-CoV-2 have also generated variants that have been hugely successful. For example, many of the favorably selected variants, such as L18F, L5F (spike); R203K, G204R, and A220V (nucleocapsid), were found to be destabilizing the respective protein structure (Table 1). As destabilizing mutations are known for their crucial functional roles, a trade-off between stabilizing and destabilizing mutations may balance the protein function and structure in ways that are not yet fully understood (33, 34).

In our study, the effect of mutations on respective proteins was primarily estimated based on the physical change in free energy for a single “native” protein conformation. To allow the most robust correlation of mutations with molecular evolution, the mutational effects for the protein in an unfolded state, and the possibility of structural adjustment of the folded state in response to the mutation, needs to be explored in future studies when more structural dynamic information becomes available (35). While our study highlights the impact of ΔΔG analyses as a reference frame for evolutionary evaluation, molecular evolution is likely a consequence of complex amalgamation of changes in free energy, entropy, solvent accessibilities, etc. (36). As the data on these unchecked parameters becomes available, predicting evolutionary selection of mutation with respect to the phylogeny would become confirmatory. Our study highlighting preliminary data linking free energy and phylogeny would help streamline the scope of future studies by providing a baseline matrix.

The currently circulating spike variants or RBD variants need to be taken into account while evaluating vaccine candidates or neutralizing monoclonal antibodies against SARS-CoV-2 (37). Mapping the viral mutations that escape antibody binding is essential for accessing the efficacy of therapeutic and prophylactic anti-SARS-CoV-2 agents (29, 38). Recently generated experimental evidence suggests that leading vaccines (mRNA-1273, BNT162b1, and ChAdOx1 nCoV-19) and two potent neutralizing antibodies (REGN10987 and REGN10933) are unlikely to be affected by the dominant variant D614G (23, 24, 39–41). As all three candidate vaccines encode RBD or the part of spike protein as antigens, the viral population is expected to try and escape by altering the positioning of the respective antigens (42) under vaccine-induced selection pressure. Notably, complete escape mutation maps of 3,804 of the 3,819 possible RBD amino acid mutations against 10 human monoclonal antibodies are already in place (29, 42). The antigenic effect of key RBD mutations against the REGN-COV2 cocktail (REGN10933 and REGN10987) showed N439K and K444R variants escaped neutralization only by REGN10987, while E406W escaped both individual REGN-COV2 antibodies and the cocktail (38). Similar strategies should be adopted to map all antibody resistance mutations against neutralizing antibodies elicited after vaccination. Once mutation escape maps are available for all successful vaccine candidates, vaccine roll-out strategies should be carefully planned to counter geographically confined escape mutants.

In conclusion, our study highlights the importance of continued genomic surveillance, mutation mapping, stability analysis, and potential escape mutation cataloguing both in the pre- and postvaccination period of SARS-CoV-2 so as to design the epidemiologically best vaccination programs. The currently observed mutation pattern and subsequent phylogenetic diversification of SARS-CoV-2 seem to be strongly influenced by the negative and positive selection pressures. The overall variation in SARS-CoV-2 sequences is currently low compared to many other RNA viruses. One of the possible reasons for the low rate of mutations can be attributed to the widespread absence of neutralizing antibodies or the selective pressure. Once the virus population is challenged with the vaccine candidates or therapeutic monoclonal antibodies, the currently known epitopes on surfaces of SARS-CoV-2 proteins are likely to undergo rapid forced change for survival. Thus, the prevalence of such possible escape mutations needs to be monitored even more carefully after vaccination if we are to remain ahead of this rapidly shifting pandemic curve.

## MATERIALS AND METHODS

### Data acquisition and curation.

In total, we have retrieved 7,000 genomes from GISAID EpiCoV database (https://www.gisaid.org/). Data sets that were flagged as complete (>28,000 bp) were screened and subsequently manually curated for excluding low quality/coverage sequences and duplicates. Sequence metadata was retrieved and only genomes containing sampling time and location were chosen for the study. Lineages were assigned from alignment file using the Phylogenetic Assignment of Named Global Outbreak LINeages tool PANGOLIN v1.07 (https://github.com/hCoV-2019/pangolin). We selected a subset of 513 genomes (Table S1 in the supplemental material) that belongs to all major PANGOLIN lineages and common mutations for the optimal output of the phylogenetic tree.

### Phylogenetic analysis.

Genome sequences were aligned against the original Wuhan-Hu-1 genome (accession: NC_045512) using multiple genome sequence alignment tool MAFFT (v6.240) (43). Subsequently, the error prone 5′-UTR and 3′-UTR regions were masked and the genome size was adjusted without losing key sites. A maximum likelihood (ML) tree was generated using IQTREE v.1.6.1 (http://www.iqtree.org/) under the GTR nucleotide substitution model with 1,000 bootstrap replicates (44). The ML tree was visualized and labeled using the interactive tree of life software iTOL v.3 (45).

### Mutation profiling.

In order to identify the genetic variants, assembled genomes were mapped against the reference (Wuhan-Hu-1: accession: NC_045512) using Snippy mapping and variant calling pipeline (https://github.com/tseemann/snippy) (46). Among the SNPs, missense SNPs (nonsynonymous) were extracted using custom-written bash scripts and manually curated as per the CoV-GLUE database (http://cov-glue.cvr.gla.ac.uk/). Specifically, we considered 11 lineage-defining mutations and 59 major missense mutations in four major structural proteins: envelope protein (E), membrane glycoprotein (M), nucleocapsid phosphoprotein (N), and spike protein (S). Structural analysis of 70 amino acid substitutions in SARS CoV-2 mutants were analyzed to examine the potential impact of these mutations on protein stability.

### Structural analysis.

The structural impact of mutations has been assessed from the COVID-3D server (http://biosig.unimelb.edu.au/covid3d), which has integrated analytics regarding mutation-based structural changes in a protein. Vibrational entropy (VE) (ΔΔS) and unfolding Gibbs free energy (FE) (ΔΔG) were considered markers to ascertain the stability of the variants. Gibbs free energy (FE) (ΔΔG) values from the site directed mutator (SDM), DUET, and DynaMut tools available in COVID-3D server were considered (47, 48). The change in vibrational entropy energy (ΔΔSVib ENCoM) between wild-type and mutant protein was calculated using DynaMut (49). VE explains the occupation probabilities of protein residues in an energy landscape based on average configurational entropies. Considerable decrease in VE increases the rigidity of the proteins (50). FE, on the other hand, describes the free energy alterations while unfolding a kinetically stable protein (49). The positive and negative values of ΔΔG indicate the stabilizing and destabilizing mutations. DynaMine (http://dynamine.ibsquare.be/) was employed to validate the stability profiles through residue level (sequence-based) dynamics. Backbone N-H S^2^ order parameter values (atomic bond vector’s movement restrictions) were generated according to the molecular reference frame. These N-H S^2^ order parameter values are evaluated from experimentally determined NMR chemical shifts. A value above 0.8 is considered highly stable, values between 0.6 and 0.8 can be considered to be functionally contextual, and values >0.6 are highly flexible (51).

### Data availability.

The genome sequences used in this study are available in the Global Initiative on Sharing All Influenza Data (GISAID) with accession IDs (see Table S1 in the supplemental material).

10.1128/mBio.01188-21.1 Figure S1 Heat map showing the stabilizing and destabilizing mutations of SARS-CoV-2 proteins based on the predicted ΔΔG values. The scale of heatmap ranged from −2 (blue) to +2 (red). Beige color in the heat map indicates neutral ΔΔG values. Download FIG S1, TIF file, 0.1 MB. Copyright © 2021 Jacob et al. 2021 Jacob et al. https://creativecommons.org/licenses/by/4.0/ This content is distributed under the terms of the Creative Commons Attribution 4.0 International license.

10.1128/mBio.01188-21.2 TABLE S1 List of SARS-CoV-2 genome sequences downloaded from GISAID with accession IDs and metadata. Download Table S1, XLSX file, 0.03 MB. Copyright © 2021 Jacob et al. 2021 Jacob et al. https://creativecommons.org/licenses/by/4.0/ This content is distributed under the terms of the Creative Commons Attribution 4.0 International license.

10.1128/mBio.01188-21.3 TABLE S2 Residue level backbone stability values of lineage-defining mutations of SARS-CoV-2. Download Table S2, XLSX file, 0.1 MB. Copyright © 2021 Jacob et al. 2021 Jacob et al. https://creativecommons.org/licenses/by/4.0/ This content is distributed under the terms of the Creative Commons Attribution 4.0 International license.
