# Supplementary figures and images for: Evolutionary Tracking of SARS-CoV-2 Genetic Variants Highlights an Intricate Balance of Stabilizing and Destabilizing Mutations
Source: mBio. 2021 Jul 20;12(4):e01188-21. doi: 10.1128/mBio.01188-21 (PMC8406184; doi:10.1128/mBio.01188-21)

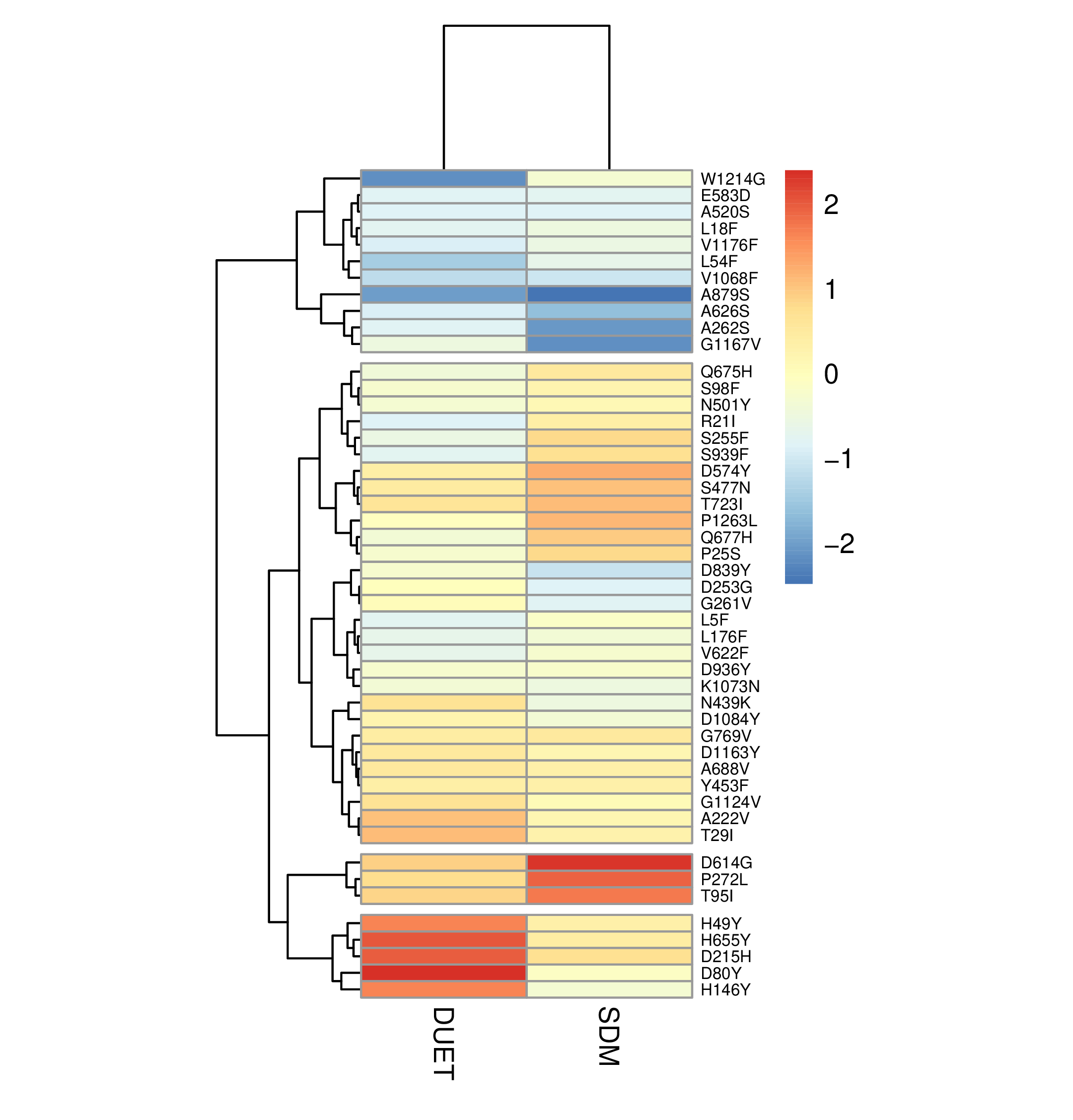

Supplement: Figure S1 [file mbio.01188-21-sf001.tif]
